# Experimental study on the eco-friendly plastic-sand paver blocks by utilising plastic waste and basalt fibers

**DOI:** 10.1016/j.heliyon.2023.e17107

**Published:** 2023-06-08

**Authors:** Bawar Iftikhar, Sophia C. Alih, Mohammadreza Vafaei, Mujahid Ali, Muhammad Faisal Javed, Usama Asif, Muhammad Ismail, Muhammad Umer, Yaser Gamil, Mugahed Amran

**Affiliations:** a School of Civil Engineering, Universiti Teknologi Malaysia, 81310, Johor Bahru Johor, Malaysia; b Department of Civil Engineering, COMSATS University Islamabad, Abbottabad Campus 22060, Pakistan; c Institute of Noise and Vibration, School of Civil Engineering, Universiti Teknologi Malaysia, 81310, Johor Bahru Johor, Malaysia; d Department of Transport Systems, Traffic Engineering and Logistics, Silesian University of Technology, Krasińskiego 8 Street, Katowice, Poland; e Department of Civil, Environmental and Natural Resources Engineering, Luleå University of Technology, Sweden; f Department of Civil Engineering, School of Engineering, Monash University Malaysia, Jalan Lagoon Selatan, 47500 Bandar Sunway, Selangor, Malaysia; g Department of Civil Engineering, College of Engineering, Prince Sattam Bin Abdulaziz University, 11942, Alkharj, Saudi Arabia; h Department of Civil Engineering, Amran University, 9677, Amran, Yemen

**Keywords:** Eco-friendly, Plastic-sand paver blocks, Plastic waste, Basalt fibers, Compressive strength, Water absorption, Temperature effect

## Abstract

Plastic waste poses a significant hazard to the environment as a result of its high production rates, which endanger both the environment and its inhabitants. Similarly, another concern is the production of cement, which accounts for roughly 8% of global CO_2_ emissions. Thus, recycling plastic waste as a replacement for cementitious materials may be a more effective strategy for waste minimisation and cement elimination. Therefore, in this study, plastic waste (low-density polyethylene) is utilised in the production of plastic sand paver blocks without the use of cement. In addition to this, basalt fibers which is a green industrial material is also added in the production of eco-friendly plastic sand paver blocks to satisfy the standard of ASTM C902-15 of 20 N/mm^2^ for the light traffic. In order to make the paver blocks, the LDPE waste plastic was melted outside in the open air and then combined with sand. Variations were made to the ratio of LDPE to sand, the proportion of basalt fibers, and sand particle size. Paver blocks were evaluated for their compressive strength, water absorption, and at different temperatures. Including 0.5% percent basalt fiber of length 4 mm gives us the best result by enhancing compressive strength by 20.5% and decreasing water absorption by 50.5%. The best results were obtained with a ratio of 30:70 LDPE to sand, while the finest sand provides the greatest compressive strength. Moreover, the temperature effect was also studied from 0 to 60 °C, and the basalt fibers incorporated in plastic paver blocks showed only a 20% decrease in compressive strength at 60 °C. This research has produced eco-friendly paver blocks by removing cement and replacing it with plastic waste, which will benefit the environment, save money, reduce carbon dioxide emissions, and be suitable for low-traffic areas, all of which contribute to sustainable development.

## 1 Introduction

**Fig. 1:**
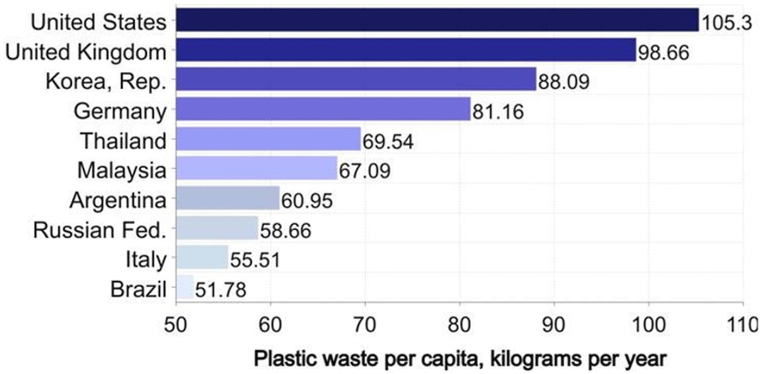
Plastic waste production per capita, per country [10].

Plastic is a very remarkable human invention; nevertheless, due to its non-biodegradable nature, it has several severe environmental effects. Plastic pollution has now become the largest substantial danger to modern society, causing environmental deterioration and economic harm [1]. The large volume of plastic debris amassing in the ecosystem has posed a threat to several marine species and environmental sustainability. Plastic waste (PW) poured into rivers and seas pollutes the water and leads to the degradation of hazardous substances contained in the plastic debris when exposed to high levels of sunlight and physical stress caused by waves [2,3]. The breakdown of plastic into minute particles also induced bioaccumulation and biomagnification in animals, which resulted in health problems for the animals [4,5]. In addition, PW can choke drainage systems, resulting in the development of parasitic insects and water-borne diseases [1], and obstructed drainage can lead to floods [6]. In addition, the large amount of PW that is routinely dumped in landfills rather than recycled has become an important incentive for developing efficient PW management procedures [7,8]. Due to the high cost and energy required for landfilling, a portion of PW accumulates or is discharged into aquatic habitats [9]. The magnitude of the plastic garbage issue is almost staggering. According to a report by Forbes in 2020, a study published in scientific advances indicates the top 10 nations that produce the most PW per person per country, as seen in Fig. 1 [10]. As plastic has a low biodegradability rate, it has contributed to the deterioration of a number of environmental issues and posed hazards to the local population.

**Fig. 2:**
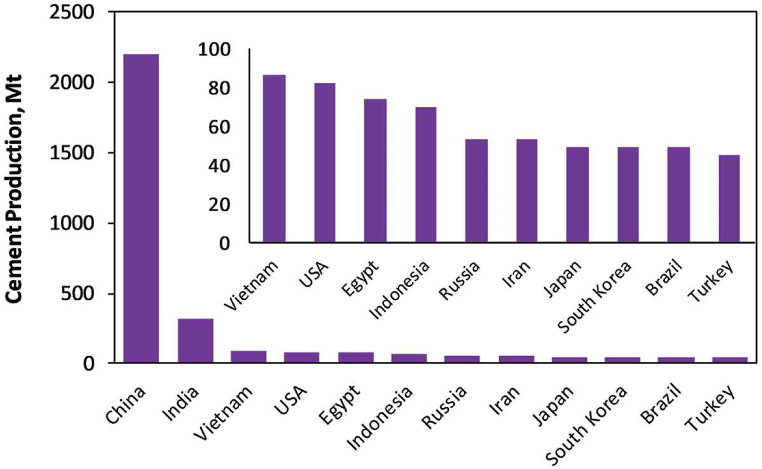
Cement output in the leading cement-producing nations in 2019 [17].

Similarly, the rising production of cement in general and the release of carbon dioxide (CO_2_) in particular pose another serious threat to the environment that environmentalists are concerned about [11,12]. To safeguard the environment, it is necessary to minimise cement use [13,14], since cement production creates an equivalent amount of CO_2_. Reducing cement consumption for cement-based goods such as mortar, concrete, and paving blocks may drastically cut CO_2_ emissions, which result in 0.9 tonnes of CO_2_ per 1 tonne of cement [15]. Cement manufacturing has accounted for approximately 8% of worldwide CO_2_ emissions [16]. The main countries involved in the production of cement in 2019 are shown in Fig. 2 [17]. It is necessary to seek substitutes to limit the consumption of cementitious materials [18,19]. To lower the carbon footprint [20] and related health issues, it is possible to substitute PW for cement as a binding medium; this will also aid in the elimination of PW. Several research and reviews on the usage of PW in the construction sector have been studied in recent years, and the findings have been promising [21,22].

The ecologically acceptable usage of PW for building purposes has both environmental and economic benefits. It has been effectively utilised to make cheaper plastic bricks [23,24]. Its use as a binder in the manufacture of paver blocks (PB) has been investigated and effectively implemented [25,26]. In comparison, PW has been incorporated into concrete blocks as a feasible and promising replacement for cement [27], fine aggregates [28], and coarse aggregates [29]. Since decades ago, concrete paver block (CPB) has gained popularity and has been extensively used in many applications such as pedestrian walks, parking areas, container yards and roads [[30], [31], [32], [33]]. Cement being the primary material and extensively used in CPB, there is a need for a reduction in the use of cement due to its emission of CO_2_ [[34], [35], [36]]. The use of cement is also time-consuming as time is delayed for the curing process to achieve its full strength [[37], [38], [39]], and also water content effects different characteristics of sand [40]. Moreover, the standard paver block requires 210 kg/m^3^ of cement, which adds to significant CO_2_ emissions [34]; therefore, there is a need to decrease cement use. One way to minimise the use of cement in PB is by its replacement with PW [23,41]. For the first time in 2006, Pierre Kamsouloum of Cameroon created plastic-bonded sand PB using recycled PW. Agyeman et al. [41] suggested that in the production of paving blocks, recycled PW can be used instead of cement as a binder. As a construction material, the usage of PW helps the environment's sustainability [9]. Similarly, with the use of the PW in the paving block, its weight is reduced to 15% than the concrete block [42]. According to the economic evaluation, the average cement-less plastic PB unit cost is 35.39% percent lower than that of the concrete block [34]. Therefore, a plastic paver is more economical in addition to the lower weight and saving our environment.

Many researchers have studied that by the addition of fibers mechanical properties of the materials are increased [[43], [44], [45], [46]]. The choice of the proper fiber depends largely on the application requirements [47]. For plastics to be used in PB, they are first melted at a temperature of 150–250 °C [26,48]. So the fiber should be selected such that it has a high melting point so that they are not damaged when they are mixed with plastic-sand (PS) in the heating process. The most commonly used fibers with high melting points along with enhanced properties are mainly carbon, glass and basalt fibers. According to a number of researches basalt fiber exhibits outstanding properties compared to other fibers [[49], [50], [51], [52]]. Basalt fibers are made from natural fibers and considered to be a green industrial material [[53], [54], [55]]. Carbon fibers have the well-known problem of being expensive; glass fibers are prone to surface degradation, whereas basalt fibers are economical and durable [56,57]. Basalt fibers are considered to be eco-friendly and have greater Young's modulus, compressive and bending strength, and energy absorption capacity than glass fiber [58]. There are no hazardous reactions between basalt products and air or water, they are non-combustible, and they are not explosive. These substances do not create any harmful chemical reactions when they come into touch with other chemicals. A risk associated with very small fibre diameters is unknown at the present time [58,59]. Thus, utilising basalt fiber in the production of PB with PW will help in environmental sustainability. Moreover, the majority of previously manufactured plastic paving blocks are adequate for pedestrian use as their compressive strength is near to or below 15 N/mm^2^ [60]. To comply with the ASTM C902-15 standard of 20 N/mm^2^ for moderate traffic [61], it is necessary to attain a higher compressive strength from plastic waste. This is achieved by the addition of basalt fibers. The present study will examine the usage of PW in the manufacturing of cement-free PB in order to investigate possible alternatives. Utilising recycled plastics for civil engineering applications reduces the environmental impact due to its long-lasting waste and ability to keep waste out of landfills. This will lead to the development of environmentally friendly PB made from PW and basalt fibers, which will help build a green construction technology.

## 2 Materials and methods

### 2.1 Low-density polyethylene (LDPE)

The LDPE used in this study was acquired from the local municipality in Abbottabad, Pakistan. After collection, the sample was first washed, and after that, it was properly cleaned and dried to eliminate any impurities that may impede the melting process, and finally, it was converted into little pieces by shredding. Table 1 shows the properties of LDPE used in this study.

**Table 1:** Characteristics of LDPE [62].

S. No	Description	Value
1	Melting point	110 °C
2	Density	0.910–0.940 gm/cm3
3	Elastic Modulus	0.6–1.4 GPa
4	Softening point	70 °C

### 2.2 Natural fine aggregates (sand)

In this particular research project, the fine aggregate was comprised of naturally occurring river sand. The characteristics of sand were determined by carrying out tests in accordance with the protocols established by the American Society of testing materials (ASTM), as shown in Table 2. The fineness modulus and specific gravity are significant factors that affect the composition's density [63]. Two types of sand (type 1 and type 2) with different fineness moduli were locally available. Sieve analysis was performed to determine the fineness modulus of both types of sand. Sand type 1 which was finer than other type was used for further study.

**Table 2:** Properties of fine aggregates.

Test	Test Results		Specifications
**Sieve analysis**	Sand type 1	Sand type 2	ASTM C136
**Specific gravity**	2.65	2.66	ASTM D854-02
**Water absorption**	4%	5.2%	ASTM C128
**Fineness Modulus**	2.9	3.2	ASTM C125

### 2.3 Basalt fiber

The current study used two lengths of 4 mm and 12 mm of basalt fibers. Among many fibers, basalt fibres (BF) are mineral fibres with superior mechanical qualities, resistance to corrosion, and compatibility [64]. Compatibility in this context refers to the increased strength of chemical bonds between the basalt fibre and the matrix, which results from their same chemical makeup [65]; the synergistic deformation capability, which reduces the pore crack damage brought on by interface fusion and the non-synchronous deformation brought on by the differences in expansion coefficients [66]; and the comparable density with concrete, which makes the basalt fibres more evenly distributed during casting [65,67]. The chemical composition of basalt fibres is shown in Table 3.

**Table 3:** The chemical composition of basalt fiber [68].

Compound	% By weight in Basalt fibres
**MgO**	1.3–3.7
**Cao**	5.2–7.8
**K**_**2**_**O**	0.8–4.5
**Fe**_**2**_**O**_**3**_	4.0–9.5
**Na**_**2**_**O**	2.5–6.4
**SiO**_**2**_	51.6–57.5
**Al2O**_**3**_	16.9–18.2

### 2.4 Sample preparation and moulding

The samples are prepared from a mix of LDPE and sand. Initially, in order to get the LDPE sample to the appropriate consistency, it is first heated up in the open air in a pan. The melted sample must be properly mixed with suitable proportions of sand. Samples in the first stage were made by using a specific PS ratio of 25%–75% with varying sand grain sizes (d < 0.42 mm, 0.42 mm < d < 0.59 mm and 0.59 mm < d < 1.68 mm). In the second stage of the research, sand grain size, which gave maximum strength, was used in different proportions of PS (20%–80%, 25%–75%, 30%–70% and 40%-60). In the third stage of research for enhancing the mechanical properties of PB, basalt fiber of lengths 4 mm and 12 mm were added in different proportions (0.1%, 0.3%, 0.5%, 0.7% and 1%) in the optimum samples. A number of 3 samples were prepared for each case; thus, the total number of samples prepared was 144. Table 4 shows variation in mix design and the respective number of samples prepared in each case.

**Table 4:** Schematic variation in the mix design of samples.

Experiment	Proportion of Plastic by weight %	Proportion of sand by weight %	Particle size of sand	Basalt Fiber Content	Compressive Strength Sample	Water Absorption Sample	At various Temperature Sample
**Effect of Particle size**	25	75	d < 0.42 mm	–	3	3	–
			0.42 mm < d < 0.59 mmm	–			
			0.59 mm < d < 1.68 mm	–			
**Effect of Various Proportions of plastic-sand**	20	80	d < 0.42 mm	–	3	3	–
	25	75		–			
	30	70		–			
	40	60		–			
Effect of basalt fiber of length 4 mm	30	70	d < 0.42 mm	0.1%	3	3	–
				0.3%			
				0.5%			
				0.7%			
				0.1%			
Effect of basalt fiber of length 12 mm	30	70	d < 0.42 mm	0.1%	3	3	–
				0.3%			
				0.5%			
				0.7%			
				1%			
Effect of basalt fiber of length 4 mm at various temperatures	30	70	d < 0.42 mm	0.5%	3	–	0 °C
							10 °C
							20 °C
							30 °C
							40 °C
							50 °C
							60 °C
Effect without basalt fiber at various temperatures	30	70	d < 0.42 mm	–	3	–	0 °C
							10 °C
							20 °C
							30 °C
							40 °C
							50 °C
							60 °C
Total Samples = 144							

**Fig. 3:**
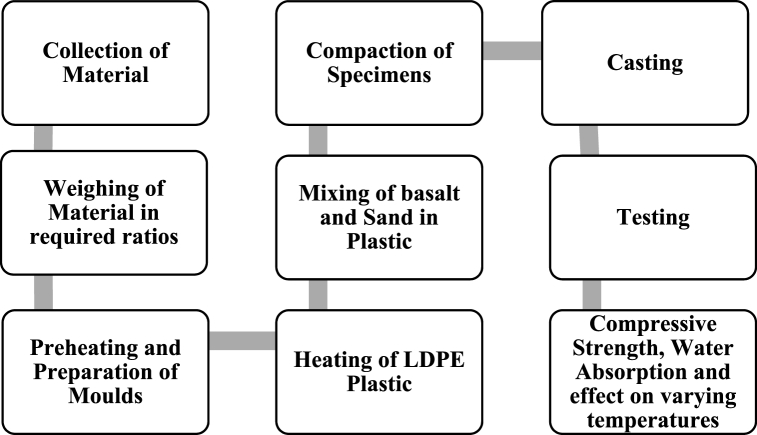
Methodology Flow diagram.

After heating of plastic calculated amount of sand and basalt fiber was added to the melted plastic and mixed thoroughly. Prior to the moulding process beginning. Heating of the moulds was done at 100 °C to make the placing and compacting of the samples in moulds easy. As if not heated, the plastic would lose its plasticity before proper compaction of sand-plastic paste. Moulds were lubricated with lubricating oil so that the samples could easily be de-moulded. The homogenous mix of plastic, fibers and sand was then poured into the pre-heated and pre-lubricated cubic mould of sizes (50 mm × 50 mm x 50 mm). These moulds were kept at room temperature for 24 h. Fig. 3 depicts the process flow diagram used to manufacture plastic sand paver blocks (PSPB).

After 24 h of moulding, 3 samples of each mix were tested for compressive strength and water absorption to get the average value. This process was followed to find the appropriate particle size of sand with a fixed PS ratio of 25%–75%. Particle grain size giving maximum strength selected in the previous stage was then used in different proportions of PS to find out the optimum ratio of PS. The optimum ratio of PS was then used with basalt fiber of 4 mm and 12 mm length with different volume content to determine the compressive strength, water absorption and also the effect of varying temperatures.

### 2.5 Experimental study

#### 2.5.1 Compressive Strength

**Fig. 4:**
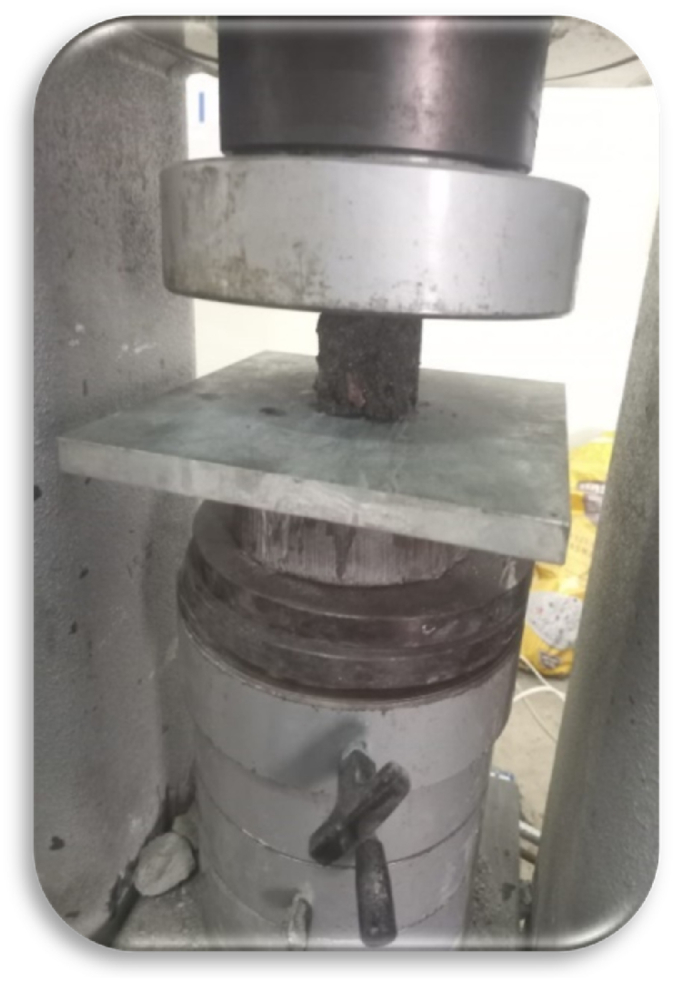
Compressive test.

Compressive Testing Machine (CTM) was used to determine the compressive strength of all the samples. Testing was done by following the testing procedure mentioned in ASTM C109/C109 M [69], which allows a loading rate at 20 MPa/s. The stress is the force applied by the CTM to a sample of a certain area. Cubes of size 50 mm × 50 mm x 50 mm were used, as shown in Fig. 4. The details about the area were provided to the CTM prior to testing. Hence CTM automatically calculates the stress taken by the sample up to failure.

#### 2.5.2 Water Absorption

Water absorption is the amount of moisture absorbed by plastic PB when placed submerged under water for about 24 h. Water absorption is important because it directly defines the durability of the sample. As per ASTM D570, the water absorption test was conducted. The samples were first dried in an oven for 24 h and weighed before placing them in the water, and the weight was named W_1_. After 24 h, samples were brought to saturated surface dried condition and weighed again; this weight was named W_2_. The water absorption was then found using Equation (1).

(1) $$Water\;absorption\;\left(\%\right)=\frac{W_{2}-W_{1}}{W_{1}}*100$$

#### 2.5.3 Temperature effects

According to the Aljazeera report, the maximum temperature of 56.7 °C around the world so far was recorded in California in 1913 [70]. Therefore, the effect at various temperatures up to 60 °C will be tested to determine the compressive strength of PB. Temperature effects were calculated by keeping the samples in the oven for 24 h at varying temperatures from 0 to 60 °C, and samples were tested at every 10 °C interval.

## 3 Results and discussion

### 3.1 Effect of sand particle size

**Fig. 5:**
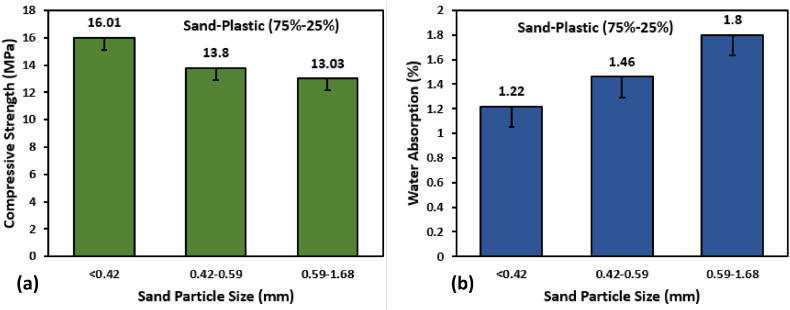
Sand Grain effect on (a) Compressive Strength and (b) Water Absorption.

The influence of sand particle size on the amount of water absorbed and the compressive strength of PSPB containing 25% by weight of LDPE PW is illustrated in Fig. 5. The sample having a particle size of diameter less than 0.42 mm shows the highest compressive strength of 16 MPa, and as the size of the sand increase, the compressive decreases up to 13 MPa. There is a negative correlation between the size of particles and compressive strength; as particle size increases, strength decreases and porosity increases [71]. And particle size with 0.59 mm < d < 1.68 mm has maximum water absorption of 1.8%. In comparison to the findings of previous studies, it was found that CPB, which was classified as hydrophilic, absorbed more water than Recycled Plastic Waste (RPW) PB, which was recognised as hydrophobic [41]. After 72 h of curing, the rate of water absorption by CPB has been reported to be 4.9% [41].

### 3.2 Effect of varying plastic-sand proportions

**Fig. 6:**
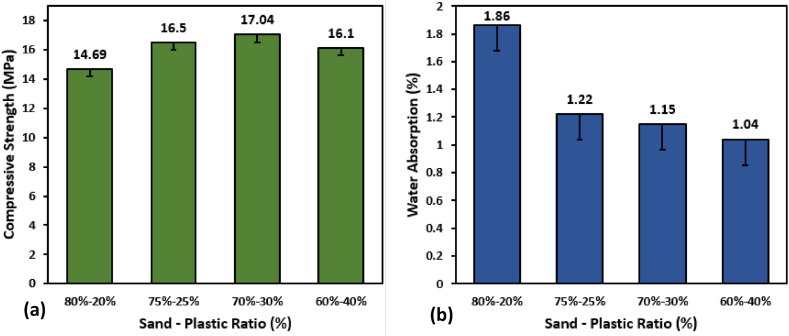
Sand-Plastic Proportions effect on (a) Compressive Strength and (b) Water Absorption.

After finding the sand particle size (d < 0.42 mm), which provided maximum strength, tests were performed to find the optimum sand-plastic ratio, which would give maximum strength. Fig. 6 shows the effect of the plastic-to-sand ratio on compressive strength and water absorption. When the proportion of plastic is low, there is an increase in water absorption and a decrease in compressive strength. Increasing the amount of plastic increases the compressive strength for additions up to 30% by weight, and after that, it starts decreasing. Maximum Compressive strength of 17 MPa was founded at a plastic-sand ratio of 30%–70%. Previous studies also showed similar results with the same ratio giving the maximum compressive strength [72]. While by increasing the plastic content, there was a decrease in water absorption was reported. Only 1.04% of water absorption was reported when 40% of plastic was used.

### 3.3 Effect of basalt fiber

**Fig. 7:**
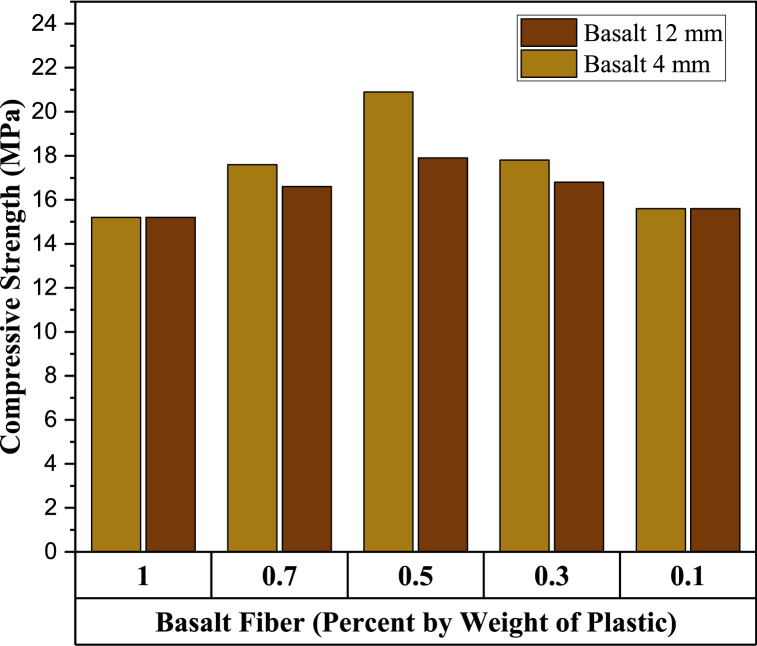
Compressive Strengths of Samples by varying percentages of Basalt fiber (lengths 4 mm and 12 mm).

**Fig. 8:**
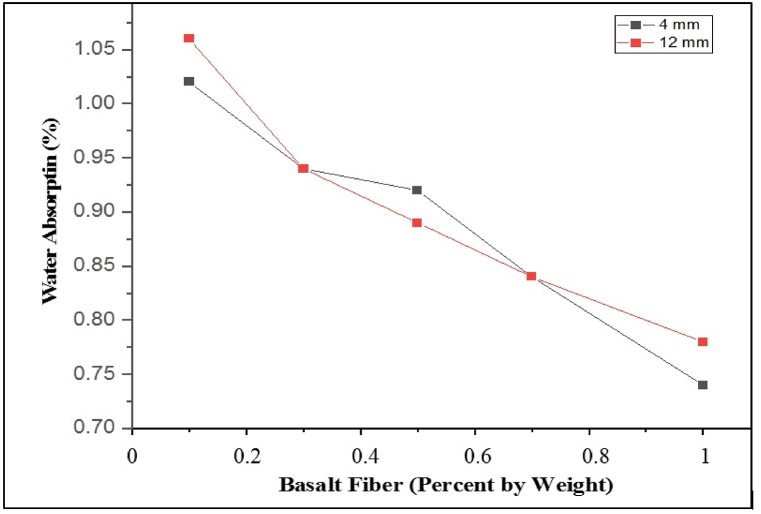
Variations of Water Absorption with varying Basalt content (4 mm and 12 mm).

Basalt fiber of different lengths and proportions was used as an additive to strengthen PB. Fig. 7, Fig. 8 show the effect of basalt fiber on compressive strength and water absorption of PSPB, respectively. Optimum plastic- Sand ratios of 30%: 70% with basalt fiber lengths of 4 mm and 12 mm were used in varying proportions of 0.1%, 0.3%, 0.5%, 1%. Results show that basalt fiber of 4 mm and 12 mm in length showed a decrease in water absorption as the content of the basalt increased, as shown in Fig. 8. In contrast, the basalt fiber of 4 mm length with 0.5% content achieved the optimum compressive strength of 21.5 MPa, while 12 mm length of basalt fiber with 0.5% content gave 18 MPa. Thus, 4 mm basalt fiber give better result by increasing compressive strength up to 20.5%, while 12 mm compressive strength has been increased up to 1%. As there is a limit to the number of fibres that can be combined, as the greater aspect ratio and longer length of fibres significantly reduces their workability, which also reduces its strength [73]. Moreover, at 7 days and 28 days, the average compressive strength of the CPB is estimated to be 10.2 MPa and 19.81 MPa, respectively [74]. Also, the requirements for the ASTM standard C902-15 [61] state that for light traffic, the compressive strength of paving brick should be atleast 20.7 MPa (average of 5) and 17.2 MPa (individual). Thus, LDPE, sand and basalt fibers can be effectively used in light traffic areas. Moreover, the 0.5% basalt fiber showed only 0.92% of water absorption which is decreased by 50.5%. Thus, incorporating basalt fibers of 4 mm about 0.5% in plastic sand PB give satisfactory result.

### 3.4 Effect of elevated temperatures

**Fig. 9:**
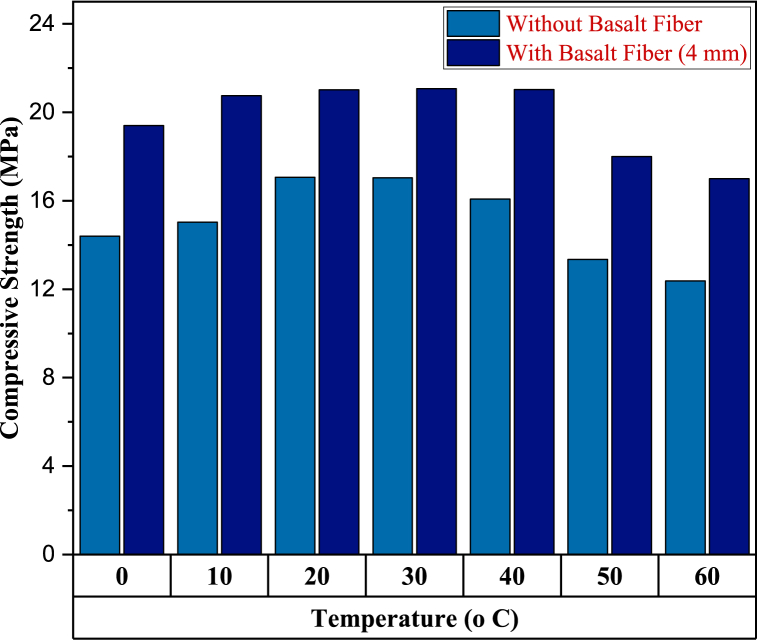
Compressive strength comparison between samples with and without basalt (4 mm) with varying temperatures.

It is predicted that samples would exhibit brittle and ductile qualities at low and high temperatures, respectively, because of the low softening and modulus of elasticity of PW. These properties will be seen at temperatures below freezing and above boiling, respectively. When plastic is the only substance used as a binding agent, the concentration of plastic used cannot be so low or so high that the material's mechanical characteristics suffer a considerable decline that is unacceptable within the margin of tolerance. In its most basic form, thermal degradation is the outcome of the manufacturing of polymers at high temperatures or their exposure to those temperatures [75]. Saxena et al. [76] also reported a decrease in compressive strength upon being heated at high temperatures. To increase the temperature resistance basalt fiber was used, as basalt has excellent thermal resistance [77,78]. The PB was prepared in the same manner with and without basalt fibers at various temperatures, i.e. 0-60 °C, as shown in Table 4. The samples of 4 mm basalt fiber with 0.5% content were only prepared, as this ratio has given us the maximum compressive strength. Results show that the temperature did not effect the PSPB having basalt fiber up to 40 °C, as basalt can preserve 90% of its typical strength even up to 600 °C [79]. While due to the excellent thermal resistance properties of basalt, the decrease was only 20% lower at 60 °C than the strength at 40 °C. Moreover, at 60 °C, the PB showed a decrease of 29.5% in compressive strength without basalt fibers, when compared with basalt fibers at the same temperature. However, a previous study reported that when PB are exposed to temperatures up to 60 °C, its strength decreases by 31.17% [34]. Similarly, at 0 °C, the compressive strength decrease was almost 30.5% when no basalt fibers were added to the PB. The effect of extreme temperatures on the compressive strength of PSPB with and without the use of basalt fiber is shown in Fig. 9.

## 4 Conclusion

This study aimed to establish a technique for converting LDPE-PW into a valuable engineering material in the shape of LDPE-bonded sand PB. This research aimed to produce PB from PW by eliminating cement. Such PB reduces the carbon footprint associated with their production by a substantial amount. This makes them a more eco-friendly alternative to conventional pavers, which typically require cement. In addition, PB is made from recycled plastic, which contributes to reducing plastic pollution in the environment. Thus, the sustainability advantages of plastic sand paver blocks make them an excellent option for paving roads, footpaths and public areas.

In this study, basalt fiber was added to the PB to enhance the compressive strength so as to meet the requirements of ASTM standard C902-15 of 20 N/mm^2^ for the light traffic. Basalt fibers were selected because they are natural fibers and have no negative effect on the environment. Thus, totally eco-friendly PB was produced from the PW, sand and basalt fibers. In addition to the compressive strength, the water absorption and temperature effect from 0 to 60 °C was also studied. The conclusion of this study is as follows.

• The LPDE-sand PB was evaluated based on the different sizes of sand particles. It was discovered that as the size of sand particles decreased, there was an increase in the compressive strength. Thus, using finer sand with a diameter of less than 0.42 mm in manufacturing LDPE-sand PB is significantly more advantageous than coarser sand.
• The effect of different LDPE-to-sand ratios on the characteristics of LDPE-sand PB was evaluated. The samples were evaluated for their compressive strength, water absorption, and effect at different temperature levels.
• The effect of basalt fibre was examined by altering its weight percentage from 0.1% to 1% of the entire mixture.
• The optimal compressive strength, water absorption, and effect at different temperatures were achieved by including up to 30% of the weight of LDPE waste in the mix. Any further addition of LDPE waste degraded the characteristics of PB.
• The 30:70 ratio of LDPE to sand produced the greatest results in terms of compressive strength (17.04 MPa) and 72-h water absorption (1.15%).
• The water absorption was drastically decreased by 50.5%–0.92% compared to the control sample.
• The most significant results were achieved by adding up to 0.5% by weight of basalt fibre of length 4 mm to the mixture. However, any addition of basalt fibre beyond 0.5% by weight of the mixture resulted in a dramatic decrease in the quality of the PB. The addition of 0.5% basalt fibre increased the compressive strength upto 21.5 MPa, which is applicable for light traffic regions.
• Moreover, the effect of temperature on LDPE-sand PB from 0 to 60 °C with and without basalt fibres at every 10 °C interval was also studied. The basalt giving the best result, i.e. 0.5% basalt fibre of length 4 mm, was used in this case. The result showed that at 0 °C (colder regions), the decrease in strength was 30.5%, while at a maximum temperature of 60 °C (very hot regions), the decrease in strength was 29.5% without basalt fibers.
• The production of PB from PW and reaching the desired strength for use in pedestrian and light traffic areas are environmentally and economically beneficial.
• Furthermore, despite being subjected to a thermal process during production, the basalt fibre pieces can fill the spaces between the particles. This characteristic permits its use to boost the mixture's density.

## 5 Limitations and future recommendations

The following are the limitations and future recommendations that can be followed for future research work in a similar field.

• This research is limited by the fact that only one basalt fiber diameter is considered. The effect of fiber diameter on the mechanical properties of PSPB must be explored further in future research.
• In our research, plastic waste was heated in an open environment; nevertheless, it is necessary to heat plastic in a controlled environment.
• The impact of using any different type of waste plastics, i.e., High-Density Polyethylene (HDPE), Medium Density Polyethylene (MDPE) and Polyethylene terephthalate (PET) in the manufacturing of plastic – sand PB should be analyzed. This will give us an insight into the different types of PW as a binding material.
• The properties of plastic–sand PB upon the addition of any other type offiber, except for basalt fiber can be examined.
• Other mechanical properties should also be studied, such as flexural strength, tensile strength and also stress-strain relationship, to know more in-depth about the plastic sand PB.

## Funding

The authors gratefully acknowledge the support given by the Lulea University of Technology, Sweden.

## Author contribution statement

Bawar Iftikhar; Sophia C. Alih; Mohammadreza Vafaei; Mujahid Ali; Muhammad Faisal Javed; Muhammad Ismail; Muhammad Umer; Yaser Gamil; Mugahed Amran: Conceived and designed the experiments; Performed the experiments; Analyzed and interpreted the data; Contributed reagents, materials, analysis tools or data; Wrote the paper.

## Data availability statement

Data included in article/supplementary material/referenced in article.

## Declaration of competing interest

The authors declare that they have no known competing financial interests or personal relationships that could have appeared to influence the work reported in this paper.
